# Analyzing the Impact of Family Structure Changes on Children’s Stress Levels Using a Stress Biomarker

**DOI:** 10.1177/00221465231223953

**Published:** 2024-02-09

**Authors:** Pauline Kleinschlömer, Mine Kühn, Lara Bister, Tobias C. Vogt, Sandra Krapf

**Affiliations:** 1 University of Mannheim, Mannheim, Germany; 2 Department of Sociology, Tilburg School of Social and Behavioral Sciences, Tilburg University, Tilburg, Netherlands; 3 Max Planck Institute for Demographic Research, Rostock, Germany; 4 University of Groningen, Groningen, Netherlands; 5 State Institute for Family Research (IFB) at the University of Bamberg, Bamberg, Bayern, Germany

**Keywords:** biomarker, child well-being, parental separation, stepfamily formation, stress

## Abstract

Changes in family structure (e.g., parental separation or stepfamily formation) are associated with a deterioration in children’s well-being. Most researchers have focused on the impact of such changes on children’s educational and psychosocial outcomes, whereas the effects on children’s biological processes have been studied less often. We analyze the effects of changes in family structure on children’s stress levels using data from the German Health Interview and Examination Survey for Children and Adolescents study (2003–2006 and 2014–2017). Our outcome variable is the biomarker c-reactive protein (CRP), which correlates with psychological distress and is collected from blood samples. Calculating first-difference estimators, we analyze whether children have higher CRP levels after changing to (1) single-parent families (n = 117) or (2) stepfamilies (n = 80). Our findings suggest that changing to a single-parent family significantly increases children’s stress, whereas changing to a stepfamily does not. These observations are important because increased stress in childhood can negatively affect well-being later in life.

The prevalence of two-biological-parent family households is declining in most Western societies, and this arrangement is increasingly being replaced by alternative family forms. In Germany, the share of underage children living with a single parent or a stepparent has risen in recent decades, from 17% among children born in 1971 to 1973 to 32% among children born in 1991 to 1993 (Kleinschlömer and Krapf 2023). Most of these children have experienced their parents’ separation (Andersson, Thomson, and Duntava 2017).

The consequences of this experience have been widely studied. Previous research suggests that on average, children living in postseparation families fare worse than children living with both biological parents (Amato 2000; Raley and Sweeney 2020) because they tend to have more behavioral and emotional problems, lower academic test scores, more problems with social relationships, and a higher risk of developing childhood obesity and asthma (Amato 2014; Bzostek and Beck 2011; Goisis, Özcan, and van Kerm 2019). Most studies attribute these adverse health outcomes to increased psychological distress because the effects of changes in family structure on a child’s personal life may lead to major adjustment problems and thus to increased psychological stress (Amato 2000). Clearly, changes in the family can also relieve stress in children, allowing them to escape the daily parental conflicts from before the separation (Booth and Amato 2001). However, on average, children’s stress increases with separation (Amato 2000).

Analyzing the association between changes in family structure and children’s stress levels is particularly important because increased stress during childhood can negatively affect many key areas of later cognitive development and physical health (Baumeister et al. 2016; Danese et al. 2009; Harkness, Bruce, and Lumley 2006). Due to a lack of data, only a few studies have used biomarkers to measure children’s stress when examining the consequences of changes in family structure even though biomarkers can serve as objective measures that reflect underlying changes in stress without any reporting bias (Eiser and Morse 2001). In particular, there is a lack of longitudinal studies on the effects of family changes on child well-being in which biomarkers have been sampled more than once over time.

This study aims to fill this gap by testing whether changes in family structure are associated with an increase in children’s stress levels using two survey waves. We are also the first to consider stepfamily formation and its consequences for children’s stress-related biomarkers in addition to studying the effects of single-parent family formation. Given that more and more children are experiencing their parents’ repartnering (Feldhaus 2016), our study makes an important contribution to the current body of research on children’s outcomes in postseparation families.

We use data from the German Health Interview and Examination Survey for Children and Adolescents (KiGGS) conducted by the Robert Koch Institute, which collected information on the health of children and adolescents living in Germany (Mauz et al. 2020; Seeling et al. 2018). These data provide information about selected biomarkers that were measured in two survey waves in 2003 to 2006 (KiGGS0) and 2014 to 2017 (KiGGS2). We use c-reactive protein (CRP) as our objective outcome variable. CRP is a biomarker of inflammatory processes and can be detected in children’s blood samples. The biomarker correlates with depressive symptoms and stress (Johnson, Abbasi, and Master 2013) and serves as a proxy for children’s stress levels in our study. There are many potential mechanisms linking changes in family structure to changes in children’s CRP, such as financial hardship or weight gain. Effects might also vary by gender or socioeconomic group. However, our aim is to analyze the direct effects of a change in family structure on children’s CRP levels in a longitudinal setting. Because CRP correlates with psychological distress, we use it as a proxy variable to infer whether children aged 1 to 17 had higher stress levels in the years after the change in family structure than in the years before.

We focus on the change from (1) a two-parent family to a single-parent family and on the change from (2) a two-parent family or a single-parent family to a stepfamily. Because of the small number of repartnering events of single parents in the first observation period, we have combined the transitions from a two-parent family and a single-parent family to a stepfamily.^1^ Of course, stepfamilies are also two-parent families. However, in our data, we cannot clarify the genetic family relationships and therefore hesitate to talk about two-biological-parent families. In our study, we define a two-parent family as a family without (reported) separation experience and define a stepfamily as a two-parent family with a history of (reported) family instability. For our analyses, we use a first-difference regression that allows us to estimate the changes in the CRP levels before and after the change in family structure within each child.

## Background

### The Impact of Changing to a Single-Parent Family on Children’s Stress Levels

A change in family structure disrupts previous family life and brings about new and potentially stressful circumstances for children. The instability hypothesis states that for children and adolescents, the departure of a parental figure creates uncertainties because they question whether they can still rely on the parent’s emotional support (Wu and Martinson 1993). Amato (2000) identified five groups of stressors that a child may experience following a parental separation: (1) financial strains; (2) parental conflicts; (3) excessive demands on the parent living with the child, which can affect the parenting style; (4) lack of contact with the nonresident parent; and (5) possible further changes in the child’s living circumstances due to moving, changing schools, or the loss of the circle of friends. Based on the instability hypothesis, we argue that children will have higher stress levels after experiencing parental separation than before (Hypothesis 1).

Previous studies have used stress-related biomarkers to analyze the effects of adverse childhood experiences on individuals’ stress levels later in life (Kuhlman et al. 2020). To our knowledge, however, only two existing studies have explicitly focused on the effects of parental separation as an adverse childhood experience (Gaydosh and Harris 2018; Lacey, Kumari, and McMunn 2013). These studies reported mixed results. Gaydosh and Harris (2018) found no effects of parental divorce in childhood on health-related biomarkers in young adulthood, specifically on CRP, metabolic syndrome, body mass index (BMI), and hypertension.^2^ By contrast, Lacey et al. (2013) found that parental separation had a positive impact on CRP in adulthood. Although both studies relied on longitudinal data to obtain information on family structure, they both measured biomarkers only once. Repeated measurements of stress-related biomarkers have been rare in previous research.

Only one study has analyzed the impact of family instability on a stress-related biomarker in children, namely, cortisol, using two stress measurements (Suor et al. 2015). The authors found that family instability predicted a stronger stress response in two-year-old children. However, they did not explicitly analyze parental separation. Instead, their definition of family instability included not only changes in the caregiver’s intimate relationship but also family events such as a change in the child’s caregiver’s job, financial losses, and the loss of a family member—with no clear distinction between these factors. For these reasons, it is difficult to derive any conclusions about children’s stress reactions to changes in family structure. In addition, the study analyzed low-income families only, which complicates the generalizability of the findings. Other longitudinal studies tried to capture stress in children following a change in family structure by measuring their adverse emotional symptoms (Cherlin, Chase-Lansdale, and McRae 1998; Rattay et al. 2018; Strohschein 2005). The questionnaires asked children whether they are currently unhappy, worried, feeling distress, or feeling anxious. The results uniformly showed that children who experienced the separation of their biological parents had lower emotional well-being than children of the same age who did not experience parental separation (Baxter, Weston, and Qu 2011; Rattay et al. 2018; Strohschein 2005).

### The Impact of Changing to a Stepparent Family on Children’s Stress Levels

A child can also experience uncertainty when a parent enters a new relationship and potentially introduces the child to a new family situation, for example to a stepfamily (Coleman, Ganong, and Fine 2000; Shafer, Jensen, and Holmes 2017; Sweeney 2010). Stepfamily formation interrupts daily routines, which can, in turn, lead to uncertainties about family roles and confusion about parenting responsibilities. In addition, research has shown that complex dynamics between half- and stepsiblings can have negative effects on a child’s well-being (Halpern-Meekin and Tach 2008). Hence, coming together as a stepfamily is a demanding and complex process that may be associated with instability and ambiguity, which could, in turn, cause children’s stress levels to rise (Coleman et al. 2000; Wu and Martinson 1993). In line with these findings, we argue that the repartnering of the resident parent can lead to an increase in children’s stress levels (Hypothesis 2).

Previous empirical studies that examined the impact of the formation of a stepfamily on children’s stress levels used mainly subjective markers. Only one study has explicitly analyzed the change to a stepfamily formation using biomarker and found no association between parental repartnering in childhood and CRP levels in young adulthood (Gaydosh and Harris 2018). Research based on subjective markers shows a positive effect of stepfamily formation on children’s stress. Hetherington and Kelly (2003) concluded that children have increased stress levels up to five to seven years after stepfamily formation. Shafer et al. (2017) showed that retrospective reports of feelings of stress after parental separation and after stepfamily formation were associated with depressive symptoms among young adults. In addition, their findings indicated that participants who perceived both parental separation and stepfamily formation in their childhood or adolescence as stressful reported higher levels of depressive symptoms than participants who perceived only one of the two changes as stressful. These results suggest that stepfamily formation may be an additional stressor on top of the stress caused by parental separation.

### Using Biomarkers to Measure Children’s Stress Responses to Changes in Family Structure

Our literature review has shown that previous studies often relied on subjective measures of children’s stress (e.g., emotional problems). However, subjective stress measurements might be biased. Whereas validated emotion self-report questionnaires are almost exclusively completed by children older than age nine, the measurement of stress in children younger than nine has been more complicated (Michels et al. 2013). In some studies, parents responded for their children. However, it has been shown that the answers of parents and children are often not identical (De Los Reyes et al. 2015). Thus, because parents’ reports are prone to reporting bias (De Los Reyes et al. 2015; Eiser and Morse 2001), using objective stress measures, such as biomarkers, has benefits when studying young children.

Research that used objective measures, including stress-related biomarkers, to study the impact of changes in family structure on children’s stress levels often relied on only one measurement of stress variables (Gaydosh and Harris 2018; Lacey et al. 2013). In such cross-sectional study designs, there is an increased risk of overlooking potential health selection effects (Gaydosh and Harris 2018). This means that isolating the impact of experiences like parental separation during childhood from other stressors becomes challenging. For instance, the parents of a child with a genetic predisposition or illness might be more likely to separate because a sick child places additional stress on the parents and on their relationship. Consequently, the differences we observe in cross-sectional studies may not solely be attributed to the experience of parental separation but to factors that are more common in families that experience parental separation. By relying on repeated measures of stress, we are able to account (at least partly) for unobserved heterogeneity in our study.

Analyzing the effects of changes in family structure on children’s stress levels is particularly important for understanding potential life course health inequalities among children living in postseparation families. Having negative experiences in childhood or adolescence may predispose individuals to later psychopathology by lowering the threshold for another stressor to be triggered in the future (Hammen, Henry, and Daley 2000; Harkness et al. 2006). The claim that such a sensitisation can occur was confirmed in the context of divorce in an experimental study by Kraft and Luecken (2009). Their study examined the extent to which young adults’ ability to cope with stress differed depending on whether they did or did not experience a parental divorce in childhood. The study found that even years after they experienced a parental divorce, the young adults’ cortisol levels showed a stronger stress reactivity response to a stressful task than the cortisol levels of young adults who did not experience a parental divorce. Therefore, we argue that children can have increased stress levels not just immediately after a change in family structure but also years after the change.

### Using the CRP to Measure Children’s Stress Levels

Following biochemical explanations, increased stress in children after a family change can be attributed to a dysregulation of the inflammatory system (Johnson et al. 2013). An inflammatory response is a natural protective reaction to a threat, such as a virus, but also to psychological or emotional stressors. The immune system releases numerous inflammatory mediators to eliminate the harmful stimuli (Herold and Mrowka 2019). A dysregulation of the inflammatory system occurs when the adaptive system is unable to resolve inflammation. As a result, further inflammatory responses are activated. The CRP, the biomarker that we use in this study, marks such reactions of the inflammatory immune system (Baumeister et al. 2016; Johnson et al. 2013). In recent years, CRP has been recognized as a significant indicator of a growing number of stress responses that are triggered by, for example, economic, social, demographic, and psychological factors (Johnson et al. 2013). There is strong evidence that adverse childhood experiences, such as changes in family structure, have a small but significant impact on children’s CRP levels, which may have long-lasting consequences for their risk of developing psychiatric and physical disorders (Baumeister et al. 2016; Kuhlman et al. 2020). Even after controlling for factors that strongly correlate with CRP, such as BMI, socioeconomic status, life events, substance use, and psychological distress, interpersonal stress involving family or friends is associated with increased CRP levels (Fuligni et al. 2009).

### Our Study

In summary, we analyze the effects of changes in family structure on children’s stress levels by measuring CRP as a stress-related biomarker in Germany. We consider the change (1) from a two-parent family to a single-parent family and the change (2) from a two-parent family or a single-parent family to a stepfamily. Previous studies that used biomarkers to investigate the effects of changes in family structure on child well-being were based on a cross-sectional research design with only one measure of the objective biomarker CRP, our proxy for stress in childhood. The uniqueness of our study is that we can rely on two measures of the objective stress marker as our outcome variable. Hence, we can add to the current literature a before–after design that more fully accounts for unobserved confounders and health selection. Previous longitudinal studies on this association often relied on subjective measures even though they are more prone to bias than objective markers. In addition, our analysis of the effects of stepfamily formation on children’s stress levels represents an important extension of previous research because much of the current research using biomarkers has analyzed only the change to a single-parent family.

Our study is based on German data. We expect that our findings can be transferred also to other countries. Like in other countries, German family patterns have become increasingly diverse during the last decades. Cohabitation is common, but the majority of couples are married (Krapf 2018), and cohabiting couples are more likely to separate than married couples (Krapf and Wagner 2020). Children experience a comparably high level of parental separation (18% of German children experience their parents separating by age 15) and repartnering (9% of children experience union formation within six years after parents’ separation; Andersson et al. 2017:1092). Although these trends in family patterns extend to other countries, there might be differences in effects of family structure. One reason for this variation could be that welfare state support for single parents differs across countries (Zagel and Hübgen 2018). Although family policies in Germany are more generous than in countries such as the United States or the United Kingdom, single mothers are much more likely to face financial burdens than coupled parents (32% of single mothers and 4% of coupled parents are poor; Härkönen 2018:41).

## Data and Method

### Data and Sample

We used data from the German KiGGS study (German Health Interview and Examination Survey for Children and Adolescents) conducted by the Robert Koch Institute (Mauz et al. 2020; Seeling et al. 2018). The data provided us with information on the health of children and adolescents living in Germany. The survey is part of the health monitoring program for children and adolescents in Germany implemented by the German Federal Ministry of Health. The KiGGS baseline study was conducted from 2003 to 2006, Wave 1 was collected from 2009 to 2012, and Wave 2 was conducted from 2014 to 2017. The survey questionnaire covered various domains of children’s physical and mental well-being. In addition, relevant demographic data and socioeconomic information on the family environment were collected. Parents were the main respondents of the KiGGS survey for children under age 11. After reaching this age, the children responded independently in the survey parts concerning them. We made use of two survey waves, KiGGS0 (2003–2006) and KiGGS2 (2014–2017), because in these waves, the questionnaire was supplemented by medical examinations, including blood sample analyses that measured the CRP. This combination of family demographic and health variables made the KiGGS data particularly suitable for our study.

In the KiGGS study, participants were recruited from 167 cities and municipalities across all German federal states with the aim of obtaining a stratified random sample of children age 0 to 17 (Mauz et al. 2020). In the baseline study (2003–2006), 17,640 participants answered the questionnaire. A total of 14,131 blood samples were provided in the baseline study of children above age 1. We imposed several restrictions on this sample to ensure that our final sample meets the theoretical and methodological requirements for our research question. First, to meet the requirements for longitudinal analysis, it is necessary to study two time points. Therefore, we restricted our sample to individuals who agreed to participate in the medical examinations twice, both in the baseline study (2003–2006) and in Wave 2 (2014–2017). This reduced our sample size to 4,743. Next, we did not consider children with missing information on relevant variables, resulting in a sample size of 1,922. Third, we considered only the 95th percentile of the CRP distribution. High CRP values indicate a likely acute infection or chronic disease rather than stress exposure, which could systematically bias our results (Sproston and Ashworth 2018). To exclude extreme values (that are most likely to be related to acute or chronic diseases), we used the distribution up to the 95th percentile.^3^ This reduced the number of cases by 194. In addition, we only kept children who were living with at least one of their parents in the same household and excluded children for whom the change to a single-parent family occurred because of the death of a parent (n = 26).^4^ Lastly, we dropped observations of children who were living in a stepfamily from the baseline KiGGS wave onward.

After applying these restrictions, we had a sample of 1,462 children ages 1 to 7 in the baseline survey (KiGGS0; 2003–2006) and ages 11 to 17 in the second wave (KiGGS2; 2014–2016). When comparing the full sample with our analytical sample, the mean values of most of our variables were similar. Age and household income were on average higher in our sample than in the full sample. This was likely related to panel attrition. Given our longitudinal study design, our sample comprised only children who participated in both KiGGGS0 and KiGGS2; those who participated only in KiGGS0 were excluded. Children who participated in KIGGS2 were by definition older than those who participated only in KiGGS0. Given that income increases with parents’ age and over calendar time, household income in the follow-up study 10 years later is higher than in the baseline study (see Appendix A in the online version of the article).

Table 1 provides a more detailed overview of the number of cases in our sample. A total of 117 children experienced a change from a two-parent family to a single-parent family between the baseline study and Wave 2. Because the number of children who experienced a change to a stepfamily between waves was limited, we considered children who experienced (a) change from a single-parent family to a stepfamily (n = 22) or (b) change from a two-parent family to a stepfamily (n = 58). A total of 80 children experienced a change to a stepfamily (Table 1), and 1,220 children continued living in a two-parent family, without any experience of family instability. This latter group did not influence the first-difference estimator. We included these children in our analyses because they serve as a useful control group for time-constant unobserved factors and allow us to obtain a more reliable estimator for the control variables (e.g., age effects; Brüderl 2010). For our second hypothesis, which focuses on switching from a two-parent family or a single-parent family to a stepfamily, children who consistently lived in a single-parent family between waves were also included in the control group (n = 45) to obtain more reliable estimators for the control variables because they were also potentially at risk of switching to a stepfamily.

**Table 1.** Descriptive Overview of Number of Children Included in the Analysis.

Family Structure (Baseline Study)	Family Structure (Wave 2)			
	Number of Children			
	Two-Biological-Parent Family	Single-Parent Family	Stepfamily	Total
Two-biological-parent family	1,220 (87.46%)	117 (8.39%)	58 (4.16 %)	1,395 (100%)
Single-parent family	0 (0.00%)	45 (67.16%)	22 (32.84%)	67 (100%)
Total	1,220 (83.45%)	162 (11.08%)	80 (5.47%)	1,462 (100%)

*Source:* German Health Interview and Examination Survey for Children and Adolescents data baseline study and Wave 2. Authors’ own calculations.

*Note:* The rows indicate the family structure of the children at the time of the baseline study. The columns indicate in which family forms the children were living in Wave 2 and thus whether the children had changed to another family form or were still living in the same family structure as in the baseline study.

### Method

To identify changes in a child’s stress-related biomarker after a change in family structure based on two time points, we used a first-difference regression. This approach estimates the effect based on a comparison of changes within an individual after he or she experienced a treatment, which is, in our case, a parental separation or a parental repartnering. Thus, we analyzed the change in a child’s stress levels from KiGGS0 to KiGGS2 while focusing on two family structure changes: the change from (1) a two-parent family to a single-parent family or the change from (2) a two-parent family or a single-parent family to a stepfamily (see Note 5 for the equation of our first-difference model).^5^ Children living continuously in a stable family structure do not contribute to the within estimator. Thereby, our analysis did not estimate the differences in children’s CRP levels between different family structures but, rather, the differences within a child’s CRP level before and after the change in family structure. We ran separate regression models for each family structure change. The resulting within estimator accounts for time-constant unobserved heterogeneity (i.e., for factors that affect both the likelihood of experiencing a change in family structure and children’s stress levels). One example of a potential unobserved time-constant confounder is children’s gender. Parents with daughters have a higher risk of separation than parents with sons (Kabátek and Ribar 2021). At the same time, girls have higher levels of CRP than boys (Cook et al. 2000). By automatically controlling for such time-constant confounders, our approach took into account the problem of omitted variables, which makes any causal claims more robust (Wooldridge 2010). Given that unobserved confounders may bias the estimated effects of changes in family structure on children’s stress levels, the first-difference design we used, unlike cross-sectional studies, can account for such biases (Ní Bhrolcháin 2001).

### Outcome Variable

Our outcome variable was children’s CRP, measured in mg/l and obtained from blood samples taken in the KiGGS baseline (2003–2006) and second wave (2014–2017).^6^ Whether a child provided a blood sample was solely based on the informed consent of the parents and not on any planned selection. We used the variable as a proxy for children’s stress levels. Other stress-related biomarkers, such as cortisol levels, were not collected in the KiGSS study. High CRP values likely indicate an acute infection or chronic disease, which may occur independently of increased distress due to family systems changes and could thus systematically bias our results (Sproston and Ashworth 2018). Because we did not have access to detailed infection-related information in KiGGS, we selected a healthy sample by excluding extreme CRP levels that were most likely associated with acute or chronic diseases. Because there is no clinically validated CRP cutoff value for acute infections or diseases in children, we selected only cases along the CRP distribution up to the 95th percentile.^7^ From the comparison of different outlier detection methods by Li, Hamann, and Beaubien (2020), percentile-based outlier removal is still one of the simplest and most effective methods for handling outliers to improve the reliability of the data. This restriction resulted in a right-skewed distribution of CRP with a maximum value of 6.37 mg/l and a mean value of .76 mg/l. Because of the skewness of the distribution, we used the log-transformed CRP variable in our regression analysis.

### Explanatory Variable

Our key explanatory variable was family structure. Because the exact dates of all family changes were not surveyed in KiGGS, we used information on the parental constellation in the child’s main residence for each wave. The variable was based on a question in which the respondent provided information on the child’s main residence at the time of the interview.^8^ The choices were whether the child was living with (1) both parents, (2) both separated parents, (3) the mother and her partner, (4) the father and his partner, (5) the mother, (6) the father, or (7) others. Using the provided information, we created our family structure variable with the following three categories: two-parent families, single-parent families, and stepfamilies. If the child was living with both parents, he or she was assigned to the “two-parent family” category. If the child was living with either the mother and her partner or the father and his partner, we operationalized the family structure as a “stepfamily.” If a child was living solely with the mother or the father, he or she was classified as living in a “single-parent family.” From the answering categories, we could not directly identify the biological parents because the category “both parents” possibly included a social parent. Genetic ties were not relevant for our study, but the number of family structure changes are. In our study, we defined a two-parent family as a family without (reported) separation experience (i.e., “both parents”) and a stepfamily as a family with a history of (reported) family instability (i.e., “the mother and her partner”).

To test our two hypotheses, we used two different family structure variables. In Hypothesis 1, we were interested in the change from a two-biological-parent family to a single-parent family. For this change, we coded a dummy variable that takes the value of 0 if the child was living in a two-parent household and the value of 1 if the child was living in a single-parent household. In Hypothesis 2, we focused on the change to a stepfamily. Again, we created a dummy variable. It took the value of 0 if the child was living in a single-parent family or a two-parent family. The variable took the value of 1 if the child had experienced a transition to a stepfamily since the last survey. In our data, we were unable to distinguish between stepchildren who were living with a single parent or with both parents at the time of the first interview. This was unproblematic with regard to the instability argument because both groups of children experienced increased instability (albeit with variation in the level of instability).

### Control Variables

We controlled for time-varying confounders by including in our regression model variables on the child’s general health status and age, the family’s socioeconomic status, and the mother’s age. An important confounder is children’s general health. Prior research has shown that children who experience a parental separation tend to have lower general health, for example, they gain more weight than children who are living with both of their parents (Goisis et al. 2019). At the same time, the CRP increases with weight (Cook et al. 2000; Ford 2003). Despite the criticism that BMI has limitations as a measure of childhood obesity because it does not fully account for growth spurts and nonlinear height and weight developments during children’s growth phases (Vanderwall et al. 2017), BMI also controls for children’s general health (Schwimmer, Burwinkle, and Varni 2003) and physiological cases of high CRPs (Cook et al. 2000; Ford 2003). For this reason, we added children’s BMI to our model as a continuous control variable, serving as a proxy for general health. The variable ranges between 12.08 and 42.44.

In addition, we controlled for the child’s socioeconomic status using the equivalent monthly household income in euros per 100 as a continuous variable. We included this control variable for two reasons: (1) because parents with fewer financial resources are more likely to separate (Amato 2010) and (2) because children living in families with fewer economic resources generally have increased health risks compared to children living in families with a higher socioeconomic status (Bradley and Corwyn 2002). The health disadvantage of individuals with a lower socioeconomic background may also be reflected in a higher CRP level (Muscatell, Brosso, and Humphreys 2020).

Moreover, we included the age of the mother in years as a categorical variable in our regression model because maternal age may be associated with health risks for the child (Carslake et al. 2017) and with the likelihood to experience changes in family structure (Lyngstad and Jalovaara 2010; Sweeney 2010). We divided the variable into three categories based on the terciles. Young mothers in the first tercile were up to 33 years old in the baseline study, 34 to 37 years comprise the middle age, and mothers over 38 years in the baseline study belong to the oldest age group. We added the age of the child as a continuous control variable to the models because CRP levels increase with age (Chiang et al. 2019; Ford 2003).

Lastly, we included the number of siblings living in the same household as the child. In single-parent families, siblings can provide each other with a safe and stable environment during the period of family structure change (Sheehan et al. 2004), which might mitigate the negative stress effects. However, in stepfamilies, complex sibship is negatively associated with child well-being (Halpern-Meekin and Tach 2008). Prior research on changes in family structure has shown that having one child decreases the risk of parental separation, whereas having additional children increases the probability of separation (Lyngstad and Jalovaara 2010).

## Results

### Descriptive Results

Table 2 summarizes the descriptive statistics for the total sample and for two-parent families, single-parent families, and stepfamilies separately. The descriptive analysis shows that in each family structure, children’s CRP values varied. Consistent with our hypothesis, we find that children living in a single-parent family had a higher CRP level (mean = .81 mg/l) than children living in a two-parent family (mean = .76 mg/l) or a stepfamily (mean = .66). Moreover, children living in a single-parent family had the lowest financial resources (equivalent monthly income [in euro per 100] mean = 12.19). Children’s financial resources increased when one parent entered a new relationship (mean = 14.52 in euro per 100), reaching a level similar to that in a two-parent family (mean = 14.57 in euro per 100). Children living in a single-parent family had a higher BMI (mean = 21.38) than children living in a stepfamily (mean = 20.83). The children’s mean age was similar in stepfamilies (mean = 14.46 years) and in single-parent families (mean = 14.96 years), and the mother’s mean age was lower in stepfamilies (mean = 42.73 years) than in single-parent families (mean = 45.75 years). Children who were living in a stepfamily had more siblings (mean = 1.86) than children who were living in a single-parent family (mean = 1.55).

**Table 2.** Descriptive Statistics for Variables Used in the Analyses in Our Samples.

Change to a Single-Parent Family (Hypothesis 1)								
Variable	Total				Single-Parent Family		Two-Biological-Parent Family	
	Mean	SD	Minimum	Maximum	Mean	SD	Mean	SD
C-reactive protein (mg/l)	.77	.99	.10	6.37	.81	.85	.76	.99
Body mass index	18.56	3.94	12.08	42.44	21.38	3.41	18.43	3.92
Net equivalent monthly household income (in euro per 100)	14.47	7.67	1.02	62.5	12.19	6.42	14.57	7.70
Mother’s age	40.00	7.27	17	60	45.75	4.83	39.75	7.26
Child’s age	9.28	5.71	1	17	14.96	1.65	9.03	5.69
Number of siblings	1.28	.96	0	9	1.55	1.07	1.27	.96
Number of observations	2,732				117		2,615	
% of observations	100				4.28		95.72	
Number of children	1,395				117		1,278	
% of children	100				8.39		91.61	
Number males	687				51		636	
% of males	100				7.42		92.58	
Number females	708				66		640	
% of females	100				9.32		90.40	
Change to a Stepfamily (Hypothesis 2)								
Variable	Total				Stepfamily		Two-Biological-Parent Family/Single-Parent Family	
	Mean	SD	Minimum	Maximum	Mean	SD	Mean	SD
C-reactive protein (mg/l)	.77	1.01	.1	6.37	.66	.89	.77	1.01
Body mass index	18.62	3.96	12.08	42.44	20.83	3.83	18.55	3.95
Net equivalent monthly household income (in euro per 100)	14.46	7.74	1.02	62.5	14.52	7.95	14.46	7.73
Mother’s age	39.94	7.30	17	60	42.73	5.18	39.86	7.34
Child’s age	9.38	5.70	1	17	14.46	1.56	9.22	5.71
Number of siblings	1.28	1.00	0	9	1.86	1.35	1.26	0.98
Number of obervations	2,690				80		2,610	
% of obervations	100				2.97		97.03	
Number of children	1,345				80		1265	
% of children	100				5.95		94.05	
Number males	665				36		629	
% of males	100				5.41		94.59	
Number females	680				44		636	
% of females	100				6.47		93.53	

*Source:* German Health Interview and Examination Survey for Children and Adolescents baseline study and Wave 2. Authors’ own calculations.

*Note:* The total columns show the descriptive statistics for the observations included in our sample for Hypothesis 1 or Hypothesis 2 (together for the baseline study and Wave 2). The single-parent family (Hypothesis 1)/stepfamily (Hypothesis 2) columns show the descriptive statistics for children who changed to a single-parent family/stepfamily between the baseline study and Wave 2. The two-biological-parent family (Hypothesis 1) and two-biological-parent family and single-parent family (Hypothesis 2) columns show the descriptive statistics for children before the change in family structure and for children who stayed in their family structure throughout the observation period. The c-reactive protein variable indicates the c-reactive protein values before the log transformation.

### Results of the Multiple Regression

**Figure 1.**
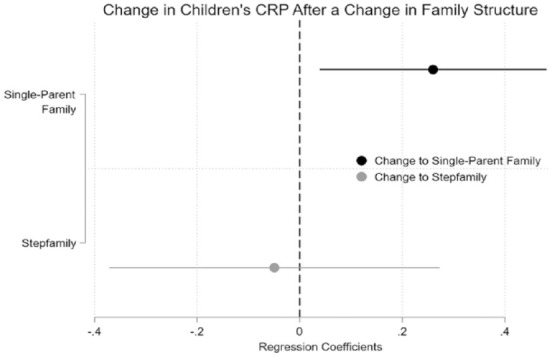
First-Difference Regression Model Results. Regression Coefficients. Outcome Variable: Children’s C-Reactive Protein (Log-Transformed). *Source:* German Health Interview and Examination Survey for Children and Adolescents (KIGGS) baseline study and Wave 2. Authors’ own calculations.

In Figure 1, we show the main effect of our two first-difference regressions. The vertical line in Figure 1 shows the average CRP level before the change in the new family structure. The gray dot represents children’s CRP level before the change to a single-parent family, and the black dot represents children’s CRP level after the change to a stepfamily.^9^ For the regression table with covariates, see Appendix B in the online version of the article.

The results support our first hypothesis. Thus, we can confirm that experiencing a parental separation led to an increase in children’s CRP levels. Children who were living in a two-parent family in the baseline study and had changed to a single-parent family in Wave 2 had a higher CRP value after the change to a single-parent family than before (β = .26, *p* = .02). However, the pattern among children who changed to a stepfamily is not as clear. Children who were living in a single-parent family or a two-parent family in the baseline study and who had changed to a stepfamily by Wave 2 had, on average, a lower CRP value than before the change in family structure. However, the effect was small (β = −.05) and was not statistically significant. Thus, this result does not support our hypothesis about stepfamily effects.

### Additional Analyses

To assess the robustness of our results, we conducted some sensitivity analyses. First, we wanted to investigate whether children who experienced their parents’ separation at an earlier point in time had adapted to the new situation. This would be demonstrated by a lower level of CRP among children who experienced their parents’ breakup earlier (e.g., soon after the baseline study). Precise separation data are not available in KiGGS. Instead, for our additional analysis, we used household composition information from the telephone survey (KiGGS1) conducted in the wave between the baseline study (KiGGS0) and Wave 2 (KiGGS2). Although no blood sample was taken in KiGGS1, information about the family structure was collected. This additional information allowed us to divide children who experienced the separation of their parents into two groups: (a) children whose parents separated before the KiGGS1 and (b) children whose parents separated after KiGGS1. For children whose parents separated before KiGGS1, there was a longer period between the separation and the second measurement of CRP levels in Wave 2 than for children whose parents separated after KiGGS1 and hence, more time to adjust to the new family structure. The results of the sensitivity analysis showed that the increase in children’s stress levels that we found in our main model was mainly caused by the children whose parents separated after the telephone interview (i.e., for whom a shorter period of time had elapsed since the separation). However, because of the small subgroups, the effect was not significant, and large confidence intervals indicate uncertainties in the results (see Appendix C in the online version of the article). Further subgroup analyses focused on the children’s age and gender. The results suggest that CRP levels increased especially among younger children and girls (see Appendices D and E in the online version of the article). However, due to our small sample size, these results should be viewed with caution because they give only a first indication of heterogeneity.

Moreover, because the two blood samples were 10 years apart, children may have experienced more than the one change in family structure that we observed in the data. To analyze the frequency of multiple family transitions after parental separation, we used the German Family Panel (2008–2021, Release 13.0; Brüderl et al. 2021). Pairfam provides partnership histories of Germans in the age group 15 to 50. It consists of a representative sample of persons born in 1971 to 1973, 1981 to 1983, and 1991 to 1993. Among single parents with minor children in the study, only 9% formed more than one new partnership within 10 years (results available on request). We therefore expect that only a small share of children experienced multiple stepfamily formations between the KiGGS survey waves.

## Discussion

Using unique information about biomarkers and family structure from the KiGGS data on children aged 1 to 17, we applied first-difference estimators to analyze the effects of changes in family structure on children’s stress levels. Specifically, we considered two separate events: (1) the change from a two-parent family to a single-parent family and (2) the change from a two-parent family or a single-parent family to a stepfamily. The biomarker CRP served as an objective measure of a proxy for stress in children. Our results indicated that children’s stress levels increased significantly after they experienced a change from a two-parent family to a single-parent family. We found no significant effects on children’s stress levels after they experienced a change from a two-biological-parent family or a single-parent family to a stepfamily.

The results of our study confirm previous findings that parental separation has adverse consequences for children. The size of the effect of separation on stress estimated in our regression analysis was β = .26. To illustrate the magnitude of the effect on CRP levels, we compare our results with those of prior research that measured stress reactions using the CRP. For example, in their study of the effects of unemployment on CRP levels in adults in the United Kingdom, Hughes et al. (2015) showed that currently unemployed individuals had a CRP level that was .22 mg/l higher than that of working individuals. Clearly, comparing these effect sizes is difficult because of differences in the samples, the research design, the age structure, the operationalization, and the measurement of the CRP variable. Nevertheless, a comparison of the effect sizes seems to indicate that the increase in CRP in the aftermath of parental separation was nonnegligible.

With regard to stepfamily formation, our results did not comply with our expectations. We hypothesized that stepfamily formation would lead to increased stress because, for example, uncertainties about family roles tend to increase after a social parent enters a joint household. Contradicting our hypothesis, our empirical analysis found no increase in children’s stress levels in response to stepfamily formation. This finding might be attributed to the considerable heterogeneity in children’s experiences of stepfamily formation. Children’s stress levels might differ depending on the time elapsed since the parental separation and the timing of the formation of the stepfamily. The KiGGS study only provides information about the family structure at the time of the survey. However, the timing of such changes can be a decisive factor because children might adapt to the new situation in the stepfamily (i.e., initial difficulties might disappear after new family roles and daily routines are established). In our study, we were unable to analyze such potential heterogeneity given the limited information in our data.

Hence, although our results showed that separation affected children’s CRP levels, we also acknowledge that the data have several limitations. Most importantly, the number of observed changes in family structure in the data was relatively small. Only 117 changes to a single-parent family and 80 changes to a stepfamily are recorded in the KiGGS data for our study population. The small sample size limited our statistical power and did not allow us to analyze heterogeneity in children’s stress responses to changes in family structure. However, heterogeneous effects (e.g., gender, child’s socioeconomic situation or age at the time of the family structure change) should be taken into account given the evidence that children do not respond identically to a change in family structure (Härkönen, Bernardi, and Boertien 2017). To assess potential moderation effects, larger longitudinal data sets that include information on children’s stress levels are required. In addition, CRP can increase for a variety of reasons (e.g., chronic stress, chronic disease, virus, or obesity). To rule out some alternative explanations, we have excluded children with a chronic disease or acute infection by omitting the 95th percentile of outliers and controlled for BMI in our regression as a proxy for children’s general health. However, we cannot fully adjudicate these different pathways. Moreover, due to sample size issues, we combined children who changed from a two-parent family to a stepfamily and children who changed from a single-parent family to a stepfamily. These children might differ in terms of the number of changes in family structure they have experienced. This could influence our results because not only the type of family structure changes but also the number of changes children experience affect their well-being (Wu and Martinson 1993).

Participation in the medical examination may pose another selectivity problem because blood sampling depends on parental consent. However, the results of a logistic regression comparing the groups of parents who did and did not give their consent uncovered no evidence of selectivity regarding family structure. Both children living in single-parent families and children living in stepfamilies were as likely to participate in the survey as children living in two-biological-parent families (see Appendix G in the online version of the article). Nevertheless, regarding stepfamilies, selection effects may have played a role in our study. It is reasonable to assume that only parents with emotionally stable children will enter a new partnership. If parents have a child who is emotionally distressed, they might decide not to enter a new relationship to avoid overwhelming the child. However, the KiGGS data do not provide information on the children’s emotional stability.

Despite these limitations, our findings are novel and contribute to the current literature on the effects of family changes on children in several ways. Our unique data from the longitudinal KiGGS study allowed us to rely on an objective biomarker as a proxy for stress levels in children: namely, the CRP obtained from the children’s blood samples. Previous research with biomarkers was mainly based on cross-sectional measurements of biomarkers in adulthood (Gaydosh and Harris 2018; Lacey et al. 2013). Our data offer the advantage of having two measurements of the biomarker in childhood. Although the measurements are 10 years apart and we do not have information about the exact timing of the family transition, we add to the literature with a short- to medium-term effect of family transitions on CRP. Although objective stress markers are associated with subjective stress measures (Michels et al. 2013), they still have an independent predictive validity (Christensen et al. 2019). Moreover, physiological measures such as CRP are more valid than self-rated stress levels because they are not subject to reporting bias. This is especially the case for younger children, who may not be able to clearly distinguish between the dimensions of stress surveyed in a questionnaire. Therefore, validated emotion self-report questionnaires are almost exclusively completed by children older than age nine (Michels et al. 2013). For younger children, researchers must rely on parental reports of child well-being. However, parents’ and children’s perspectives do not always align (De Los Reyes et al. 2015; Eiser and Morse 2001). Our study was able to circumvent this bias by relying on a biomarker as a proxy for child stress.

Moreover, our use of an objective measure of stress implies that parental separation will have long-term effects on children. Prior research indicates that elevated CRP levels in children and adolescents are associated with an increased later-life risk of developing cardiovascular diseases (Cook et al. 2000; Ford 2003; Fuligni et al. 2009), higher BMI and obesity (Cook et al. 2000; Ford 2003; Nappo et al. 2013), and depressive episodes (Danner et al. 2003). From this perspective, our findings underline the value of studying the impact of changes in family structure during childhood on children’s stress levels, which might have implications for their health later in life.

In addition, we are among the first to analyze the effects of parental separation on children using a biomarker to measure children’s stress levels before and after a family structure change has taken place. Prior research that examined the effects of changes in family structure on biomarkers have relied on only one measurement of the biomarker (Gaydosh and Harris 2018; Lacey et al. 2013). Whereas in Great Britain, experiencing parental divorce in childhood was associated with higher levels of CRP in middle adulthood (Lacey et al. 2013), this was not found in the United States (Gaydosh and Harris 2018). By contrast, in the United States, children of instable families had slightly lower risks of hypertension and metabolic syndrome than those in stable two-biological-parent families (Gaydosh and Harris 2018). With a focus on short- to medium-term effects in our analysis, we were able to identify negative effects of separation (but not stepfamily formation) on children in Germany. Most importantly, the KiGGS data include two CRP measurements during childhood and adolescence, enabling us to reduce the influence of potential selection effects. Given that child health might affect both parents’ partnership decisions and CRP levels, the first-difference model avoids bias related to such unobserved heterogeneity. Moreover, as family structures become more diverse, it is important that this diversity is reflected in research. We contributed to this research need by analyzing the effects of repartnering on children. Previous studies mainly focused on the effects of parental separation and less on the effects of stepfamily formation. Thus, our study provides further insights into the impact of changes in family structure on children’s stress levels.

While taking all the limitations and strengths of our study into account, our results have implications for policymakers and point to directions for future research on the effects of changing family structures on stress levels in children. Policymakers in Germany are mainly concerned with addressing the needs of adults after they divorce or separate (e.g., through monetary benefits, access to the labor market; BMFSFJ - Federal Ministry for Family Affairs, Senior Citizens, Women and Youth 2021). Based on our findings, we urge policymakers to consider more seriously the effects of parental separation on children’s stress levels in order to reduce inequalities. For example, one promising approach to promoting the healthy regulation of children’s physiological stress response systems after adverse childhood experiences is providing children with psychosocial support (Slopen, McLaughlin, and Shonkoff 2014).

Future research should complete the picture of stress in children by considering both subjective and objective markers of stress as dependent variables in order to compare the stress-related effects of the two measures on children. From a methodological point of view, this would show the overlap of the two measurement variants and provide information about their robustness. Future studies of larger data sets should take into consideration potential effect heterogeneity. This might also help to identify groups who are unaffected by their parents’ separation and repartnering. As a starting point, our additional analyses on gender and age can serve as a reference (see Appendices D and E in the online version of the article). Although based on very small sample sizes, the results from these analyses indicate that CRP increases especially for girls and young children. In terms of heterogeneity, it would also be interesting to analyze in future research how stress develops in the years after separation. Do stress levels remain high, or do they decrease in the years following separation? However, addressing this question would require researchers to measure the objective stress marker at more than two points in time. In addition, there are many potential mechanisms linking changes in family structure to changes in children’s CRP (e.g., monetary resources, moving, interparental stress, contact with the nonresident parent, weight gain). Deciphering these mechanisms behind increased stress levels would allow researchers to improve our understanding of children’s stress levels after they experience a change to a single-parent family.

## Supplemental Material

sj-docx-1-hsb-10.1177_00221465231223953 – Supplemental material for Analyzing the Impact of Family Structure Changes on Children’s Stress Levels Using a Stress Biomarker Supplemental material, sj-docx-1-hsb-10.1177_00221465231223953 for Analyzing the Impact of Family Structure Changes on Children’s Stress Levels Using a Stress Biomarker by Pauline Kleinschlömer, Mine Kühn, Lara Bister, Tobias C. Vogt and Sandra Krapf in Journal of Health and Social Behavior

## Notes

1. Based on the literature, we expect that both separation and repartnering disrupt daily routines and lead to uncertainty about family roles, which should cause children’s stress levels to increase. To reduce heterogeneity across children’s experiences of forming a stepfamily, we conducted a separate analysis only for children who reported living in a two- parent family in the baseline study and in a stepfamily in Wave 2. We replicated our main findings in the subgroup analysis (see Appendix F in the online version of the article).

2. Gaydosh and Harris (2018) estimated also models where they used a composite measure of instability based on the count of parents’ partnership transitions but also parental death, incarceration, adoption, and fosterage. Their results showed that instability during childhood was negatively related to hypertension and metabolic syndrome in adult ages. The authors suggest that such protective effects of family transitions may occur especially in conflictual families where a change relieves stress in children. Another explanation would be that children in instable families develop strategies that support their well-being or buffer against stress exposure.

3. The results were robust regardless of whether we used the 90th or 95th percentile (see Appendix I in the online version of the article). To keep more observations, we utilized the 95th percentile of the distribution as our cutoff point.

4. The vast majority of children in our sample who were living in postseparation families were living with their mother (93.20%), which is in line with the official statistics. However, for sample size reasons, we also included in our sample children who were living with their father. Sensitivity analyses showed that the results were robust when we considered only children who were living with their biological mother (see Appendix H in the online version of the article).

5. The equation of our first-difference regression underlines our analytical strategy:
$\Delta y_{it}=\theta \Delta d_{it}\times \beta \Delta X_{it}+\Delta \varepsilon_{it},$ where i is the index for individuals; t is the index for the time points $(t_{1},t_{2});$ ∆ $y_{it}$ is the first-differenced outcome variable, children’s CRP (difference between $t_{1}$ and $t_{2});$ θ is our treatment variable coefficient, the change in family structure; ∆ $d_{it}$ is the difference in the treatment variable d between $t_{1}$ and $t_{2};$ β∆ $X_{it}$ is the matrix of our first-differenced covariates; and ∆ $\varepsilon_{it}$ is our first-differenced error term.

6. In the baseline study of KiGGS (2003–2006), two different companies were involved in the CRP measurement from the blood samples: ROCHE and SCIL. Their measurement procedures differed, but the CRP values were converted to make them comparable (Truthmann et al. 2012).

7. In additional analyses (available on request), we excluded children above the 96th percentile, 97th percentile, and so on. With the respective samples, the coefficients of the variable *change to single-parent family* in our regression analyses were not statistically significant at *p* = .05. One reason for this might be that keeping children with very high CRP levels in the sample biased our results. This interpretation was supported when using more conservative cutoff values, (e.g., based on the 90th percentiles): In this sample, the point estimate of the variable *change to single-parent family* was statistically significant (*p* < .05; see Appendix I in the online version of the article).

8. Kuhlemann and Krapf (2022) have shown that the formation of a nonresidential partnership can also affect children’s well-being. However, in KiGGS, information about parents’ relationships with partners living in separate households was unavailable.

9. The effects are already significant at *p* = .1 in the null model, in which we analyze the relationship between family changes and CRP without any control variables. The addition of our controls increases the effect size and leads to a significance level of *p* = .05 (see Appendix J in the online version of the article).
