# Supplementary material for: Analyzing the Impact of Family Structure Changes on Children’s Stress Levels Using a Stress Biomarker
Source: J Health Soc Behav. 2024 Feb 9;65(3):449–65. doi: 10.1177/00221465231223953 (PMC11380358; doi:10.1177/00221465231223953)
Supplement: sj-docx-1-hsb-10.1177_00221465231223953 – Supplemental material for Analyzing the Impact of Family Structure Changes on Children’s Stress Levels Using a Stress Biomarker [file sj-docx-1-hsb-10.1177_00221465231223953.docx]

**Journal** of **Health**

and **Social Behavior**

OFFICIAL JOURNAL OF THE AMERICAN SOCIOLOGICAL ASSOCIATION

**ONLINE SUPPLEMENT**

**to article in**

Journal of Health and Social Behavior

**Analyzing the Impact of Family Structure Changes on Children’s Stress Levels Using a Stress Biomarker**

**Pauline Kleinschlömer**

*University of Mannheim*

**Mine Kühn**

*Tilburg University*

**Lara Bister**

*University of Groningen*

**Tobias C. Vogt**

*University of Groningen*

**Sandra Krapf**

*University of Bamberg*

Appendix A

**Table 1:** Comparison of our key variables in our original sample and final sample.

|  | Original Sample | | Final Sample | |
| --- | --- | --- | --- | --- |
|  | Mean | SD | Mean | SD |
| Age of Child | 13.04 | 6.69 | 9.39 | 5.70 |
| Net equivalent monthly household income [in Euro per 100] | 13.00 | 7.31 | 14.26 | 7.64 |
| Age of Mother | 41.97 | 7.79 | 39.97 | 7.30 |
| Number of Siblings | 1.06 | 1.15 | 1.29 | 1.00 |
| BMI | 19.53 | 4.43 | 18.61 | 3.95 |
| CRP | 1.38 | 3.77 | 0.76 | 0.99 |

*Source*: KiGGS data baseline study and wave 2. Author's own calculations.

Note: The table includes joint information for the sample for hypothesis 1 and hypothesis 2. Hence, it includes joint information on children living in two-parent families, single-parent families, and stepfamilies.

Appendix B

**Table 2:** Linear first-difference regression model results.

|  | (1) | (2) |
| --- | --- | --- |
|  | C-reactive protein | C-reactive protein |
| Family structure change |  |  |
| No change | 0 |  |
| Change to a single-parent family | 0.26* |  |
|  | (0.02) |  |
| No change |  | 0 |
| Change to a stepfamily |  | -0.05 |
|  |  | (0.76) |
| Child’s age | -0.07*** | -0.07*** |
|  | (0.00) | (0.00) |
| Body Mass Index | 0.13*** | 0.13*** |
|  | (0.00) | (0.00) |
| Equivalent monthly household -----income [in euro per 100]  Mother’s age | 0.01 | -0.01 |
|  | (0.24) | (0.21) |
| 17 – 33 years | 0 | 0 |
| 34 – 37 years | -0.11 | -0.1 |
|  | (0.14) | (0.22) |
| 38 – 60 years | -0.11 | -0.12 |
|  | (0.17) | (0.17) |
| Number of siblings | -0.04 | -0.04 |
|  | (0.37) | (0.40) |
| Constant | -2.56*** | -2.57*** |
|  | (0.00) | (0.00) |
| Observations | 2754 | 2695 |
| Adj. R^2^ | 0.11 | 0.11 |
| *p-values are in parentheses* | | |
| **** p<.001, ** p<.01, * p<.05* | | |
|  | | |

Regression coefficients. Outcome variable: children's c-reactive protein (log-transformed). *Source*: KiGGS data baseline study and wave 2. Author's own calculations.

Appendix C

|  | Separation before wave 1 | | Separation after wave 1 | |
| --- | --- | --- | --- | --- |
|  | (3) | (4) | (5) | (6) |
|  | C-reactive protein | C-reactive protein | C-reactive protein | C-reactive protein |
| Family structure change |  |  |  |  |
| No change | 0 |  | 0 |  |
| Change to a single-parent family | -0.13 |  | 0.22 |  |
|  | (0.80) |  | (0.12) |  |
|  |  |  |  |  |
| No change |  | 0 |  | 0 |
| Change to a stepfamily |  | 0.23 |  | 0.12 |
|  |  | (0.60) |  | (0.54) |
| Child’s age | -0.07*** | -0.07*** | -0.07*** | -0.07*** |
|  | (0.00) | (0.00) | (0.00) | (0.00) |
| Body Mass Index | 0.14*** | 0.14*** | 0.13*** | 0.14*** |
|  | (0.00) | (0.00) | (0.00) | (0.00) |
| Equivalent monthly household income  [in euro per 100] | 0.01 | 0.01 | 0.01 | 0.01 |
|  | (0.26) | (0.23) | (0.25) | (0.28) |
| Mother’s age |  |  |  |  |
| 17 – 33 years | 0 | 0 | 0 | 0 |
| 34 – 37 years | -0.10 | -0.10 | -0.13* | -0.12 |
|  | (0. 20) | (0.21) | (0.09) | (0.13) |
| 38 – 60 years | -0.09 | -0.09 | -0.08 | -0.10 |
|  | (0.31) | (0.30) | (0.34) | (0.24) |
| Number of siblings | -0.04 | -0.04 | -0.06 | -0.03 |
|  | (0.36) | (0.32) | (0.18) | (0.44) |
| Constant | -2.65*** | -2.64*** | -2.56*** | -2.65*** |
|  | (0.00) | (0.00) | (0.00) | (0.00) |
| Observations | 2491 | 2497 | 2638 | 2598 |
| Adj R^2^ | 0.12 | 0.12 | 0.11 | 0.12 |
| *p-values are in parentheses* | | |  |  |
| **** p<.01, ** p<.05, * p<.1* | | |  |  |
|  | | |  |  |

**Table 3:** First-difference regression model results depending on the time of the change in family structure.

Regression coefficients. Outcome variable: children's c-reactive protein (log-transformed). *Source*: KiGGS data baseline study and wave 2. Author's own calculations.

Appendix D

**Table 4:** First-difference regression model results by gender. Regression coefficients.

|  | Male | | Female | |
| --- | --- | --- | --- | --- |
|  | (7) | (8) | (9) | (10) |
|  | C-reactive protein | C-reactive protein | C-reactive protein | C-reactive protein |
| Family structure change |  |  |  |  |
| No change | 0 |  | 0 |  |
| Change to a single-parent family | 0.02 |  | 0.44** |  |
|  | (0.89) |  | (0.01) |  |
|  |  |  |  |  |
| No change |  | 0 |  | 0 |
| Change to a stepfamily |  | -0.30 |  | 0.18 |
|  |  | (0.22) |  | (0.41) |
| Child’s age | -0.05*** | -0.05*** | -0.09*** | -0.09*** |
|  | (0.00) | (0.00) | (0.00) | (0.00) |
| Body Mass Index | 0.11*** | 0.11*** | 0.15*** | 0.15*** |
|  | (0.00) | (0.00) | (0.00) | (0.00) |
| Equivalent monthly household income  [in euro per 100] | 0.002 | 0.002 | 0.01 | 0.01 |
|  | (0.76) | (0.73) | (0.2) | (0.17) |
| Mother’s age  17 – 33 years |  |  |  |  |
|  |  |  |  |  |
| 34 – 37 years | -0.20* | -0.22* | -0.10 | -0.001 |
|  | (0.05) | (0.03) | (0.34) | (0.99) |
| 38 – 60 years | -0.16 | -0.18 | -0.13 | -0.09 |
|  | (0.14) | (0.12) | (0.29) | (0.48) |
| Number of siblings | -0.03 | -0.03 | 0.04 | -0.05 |
|  | (0.66) | (0.66) | (0.52) | (0.43) |
| Constant | -2.40*** | -2.42*** | -2.84*** | -2.77*** |
|  | (0.00) | (0.00) | (0.00) | (0.00) |
| Observations | 1396 | 1333 | 1448 | 1362 |
| Adj R^2^ | 0.11 | 0.12 | 0.12 | 0.13 |
| *p-values are in parentheses* | | |  |  |
| **** p<.001, ** p<.01, * p<.05* | | |  |  |
|  | | |  |  |

Outcome variable: children's c-reactive protein (log-transformed). Source: KiGGS data baseline study and wave 2. Author's own calculations.

Regression coefficients. Outcome variable: children's c-reactive protein (log-transformed). Source: KiGGS data baseline study and wave 2. Author's own calculations.

Appendix E

|  | Children >=6 years old | | Children <=15 years old | |
| --- | --- | --- | --- | --- |
|  | (11) | (12) | (13) | (14) |
|  | C-reactive protein | C-reactive protein | C-reactive protein | C-reactive protein |
| Family structure change |  |  |  |  |
| No change | 0 |  | 0 |  |
| Change to a single-parent family | 0.49* |  | 0.07 |  |
|  | (0.02) |  | (0.70) |  |
|  |  |  |  |  |
| No change |  | 0 |  | 0 |
| Change to a stepfamily |  | -0.26 |  | -0.11 |
|  |  | (0.38) |  | (0.66) |
| Child’s age | -0.03 | -0.03 | -0.08*** | -0.08*** |
|  | (0.08) | (0.12) | (0.00) | (0.00) |
| Body Mass Index | 0.10*** | 0.10*** | 0.13*** | 0.13*** |
|  | (0.00) | (0.00) | (0.00) | (0.00) |
| Equivalent monthly household income  [in euro per 100] | 0.02 | 0.02 | 0.0003 | 0.001 |
|  | (0.15) | (0.11) | (0.97) | (0.92) |
| Mother’s age |  |  |  |  |
| 17 – 33 years |  |  |  |  |
| 34 – 37 years | 0.01 | 0.09 | -0.08 | -0.03 |
|  | (0.97) | (0.69) | (0.51) | (0.81) |
| 38 – 60 years | -0.03 | 0.03 | -0.16 | -0.15 |
|  | (0.87) | (0.91) | (0.24) | (0.29) |
| Number of siblings | -0.04 | -0.05 | 0.10 | -0.01 |
|  | (0.68) | (0.70) | (0.20) | (0.82) |
| Constant | -2.77*** | -2.71*** | -2.59*** | -2.51*** |
|  | (0.00) | (0.00) | (0.00) | (0.00) |
| Observations | 1700 | 1604 | 2012 | 1923 |
| Adj R^2^ | 0.16 | 0.14 | 0.17 | .18 |
| *p-values are in parentheses* | | |  |  |
| **** p<.001, ** p<.01, * p<.05* | | |  |  |

**Table 5:** First-difference regression model results by age groups.

|  | (15) |
| --- | --- |
|  | C-reactive protein |
| Family structure change |  |
| No change | 0 |
| Change to a stepfamily | -0.05 |
|  | (0.76) |
| Child’s age | -0.07*** |
|  | (0.00) |
| Body Mass Index | 0.13*** |
|  | (0.00) |
| Equivalent monthly household income  [in euro per 100] | -0.01 |
|  | (0.21) |
| Mother’s age  17 – 33 years |  |
|  | 0 |
| 34 – 37 years | -0.10 |
|  | (0.21) |
| 38 – 60 years | -0.12 |
|  | (0.17) |
| Number of siblings | -0.04 |
|  | (0.40) |
| Constant | -2.57*** |
|  | (0.00) |
| Observations | 2673 |
| Adj R^2^  *p-values are in parentheses*  **** p<.001, ** p<.01, * p<.05* | 0.11 |

Appendix F

**Table 6:** First-difference regression model for children who changed from a two-parent family to a stepfamily.

Regression coefficients. Outcome variable: children's c-reactive protein (log-transformed). Source: KiGGS data baseline study and wave 2. Author's own calculations.

Appendix G

**Table 7:** Logistic regression to analyse selectivity of the sample.

|  | (16) |
| --- | --- |
|  | Blood sample |
| Main residence: |  |
| Two-biological-parent family | 0 |
| Single-parent family | 0.37 |
|  | (0.24) |
| Stepfamily | 0.01 |
|  | (0.99) |
| Sex | -0.03 |
|  | (-0.86) |
| Age at survey in years | 0.04*** |
|  | (0.000) |
| Equivalent monthly household income  [in euro per 100] | 0.02  (0.15) |
| Father's highest education (ISCED) | 0.20 |
|  | (0.17) |
| Constant | 2.17*** |
|  | (0.000) |
| Observations | 6242 |
| Pseudo R^2^ | 0.01 |
| *p-values are in parentheses* | |
| **** p<.001, ** p<.01, * p<.05* | |
|  | |

Regression coefficient. Outcome variable: dummy indicating whether children’s blood sample is available. Source: KiGGS data baseline study and wave 2. Author's own calculations.

|  | (17) | (18) |
| --- | --- | --- |
|  | C-reactive protein | C-reactive protein |
| Family structure change |  |  |
| No change | 0 |  |
| Change to a single-parent family | 0.27* |  |
|  | (0.02) |  |
| No change |  | 0 |
| Change to a stepfamily |  | -0.08 |
|  |  | (0.63) |
| Child’s age | -0.07*** | -0.07*** |
|  | (0.00) | (0.00) |
| Body Mass Index | 0.13*** | 0.13*** |
|  | (0.00) | (0.00) |
| Equivalent monthly household income [in euro per 100) | 0.01  (0.33) | 0.01  (0.22) |
| Mother’s age  17 – 33 years |  |  |
|  | 0 | 0 |
| 34 – 37 years | -0.13 | -0.09 |
|  | (0.07) | (0.22) |
| 38 – 60 years | -0.13 | -0.11 |
|  | (0.12) | (0.19) |
| Number of siblings | 0.01 | -0.04 |
|  | (0.75) | (0.38) |
| Constant | -2.57*** | -2.57*** |
|  | (0.00) | (0.00) |
| Observations | 2830 | 2688 |
| Adj. R^2^ | 0.11 | 0.11 |
| *p-values are in parentheses* | | |
| **** p<.001, ** p<.01, * p<.05* | | |
|  | | |

Appendix H

**Table 8:** First-difference regression model for children who were living with their biological mother in a single-parent family or a stepfamily.

Regression coefficients. Outcome variable: children's c-reactive protein (log-transformed). Source: KiGGS data baseline study and wave 2. Author's own calculations.

Appendix I

**Table 9:** In the first-difference regression model results presented here, the upper 90th percentile is excluded from the CRP variable.

|  | (19) | (20) |
| --- | --- | --- |
|  | C-reactive protein | C-reactive protein |
| Family structure change |  |  |
| No change | 0 |  |
| Change to a single-parent family | 0.22* |  |
|  | (0.05) |  |
| No change |  | 0 |
| Change to a stepfamily |  | 0.70 |
|  |  | (0.66) |
| Child’s age | -0.06*** | -0.06*** |
|  | (0.00) | (0) |
| Body Mass Index | 0.11*** | 0.11*** |
|  | (0.00) | (0.00) |
| Equivalent monthly household income [in euro per 100] | 0.01 | 0.01 |
|  | (0.08) | (0.06) |
| Mother’s age  17 – 33 years |  |  |
|  | 0 | 0 |
| 34 – 37 years | -0.07 | -0.04 |
|  | (0.34) | (0.57) |
| 38 – 60 years | -0.10 | -0.11 |
|  | (0.18) | (0.18) |
| Number of siblings | -0.06 | -0.08 |
|  | (0.13) | (0.06) |
| Constant | -2.49*** | -2.51*** |
|  | (0.00) | (0.00) |
| Observations | 2454 | 2396 |
| Adj. R^2^ | 0.10 | 0.11 |
| *p-values are in parentheses* | | |
| **** p<.001, ** p<.01, * p<.05* | | |
|  | | |

Regression coefficients. Outcome variable: children's c-reactive protein (log-transformed). Source: KiGGS data baseline study and wave 2. Author's own calculations.

Appendix J

**Table 10:** First-difference regression results showing the null model and the successive addition of the control variables.

|  | Change to a single-parent family | | | | | Change to a stepfamily | | | | | |
| --- | --- | --- | --- | --- | --- | --- | --- | --- | --- | --- | --- |
|  | (21) | (22) | (23) | (24) | (25) | (26) | (27) | | (28) | (29) | (30) |
|  | Null model | +  Household Income | + age (mother + child) | + siblings | +  Body Mass Index | Null model | +  Household Income | | + age  (mother + child) | + siblings | +  Body Mass Index |
| Family structure changes |  |  |  |  |  |  |  | |  |  |  |
| No change | 0 | 0 | 0 | 0 | 0 |  |  | |  |  |  |
| Change to a single-parent family | 0.18 | 0.18 | 0.25* | 0.25* | 0.26* |  |  | |  |  |  |
|  | (0.10) | (0.10) | (0.03) | (0.03) | (0.02) |  |  | |  |  |  |
| No Change |  |  |  |  |  | 0 | 0 | | 0 | 0 | 0 |
| Change to a stepfamily |  |  |  |  |  | -0.27 | -0.27 | | -0.19 | -0.16 | -0.05 |
|  |  |  |  |  |  | (0.09) | (.10) | | (0.25) | (0.34) | (0.76) |
| Net equivalent household income (in euro per 100) |  | -0.002 | 0.003 | 0.002 | 0.01 |  | -0.002 | | 0.002 | 0.002 | 0.01 |
|  |  | (0.61) | (0.62) | (0.67) | (0.24) |  | (0.58) | | (0.65) | (0.70) | (0.21) |
| Age child |  |  | -0.003 | -0.001 | -0.07*** |  |  | | -0.001 | 0.001 | -0.07*** |
|  |  |  | (0.69) | (0.90) | (0.00) |  |  | | (0.88) | (0.89) | (0.00) |
| Age of mother |  |  |  |  |  |  |  | |  |  |  |
| 17 – 33 years |  |  | 0 | 0 | 0 |  |  | | 0 | 0 | 0 |
| 34 – 37 years |  |  | -0.12 | -0.11 | -0.11 |  |  | | -0.12 | -0.10 | -0.10 |
|  |  |  | (0.13) | (0.19) | (0.14) |  |  | | (0.15) | (0.22) | (0.21) |
| 38 – 60 years |  |  | -0.12 | -0.11 | -0.11 |  |  | | -0.14 | -0.13 | -0.12 |
|  |  |  | (0.18) | (0.21) | (0.17) |  |  | | (0.12) | (0.15) | (0.17) |
| Number of siblings |  |  |  | -0.05 | -0.04 |  |  | |  | -0.06 | -0.04 |
|  |  |  |  | (0.25) | (0.37) |  |  | |  | (0.21) | (0.40) |
| Body Mass Index |  |  |  |  | 0.13*** |  |  | |  |  | 0.13*** |
|  |  |  |  |  | (0.00) |  |  | |  |  | (0.00) |
| Constant | -0.83*** | -0.80*** | -0.75*** | -0.70*** | -2.56*** | -0.82*** | -0.79*** | | -0.74*** | -0.69*** | -2.57*** |
|  | (0.00) | (0.00) | (0.00) | (0.00) | (0.00) | (0.00) | (0.00) | | (0.00) | (0.00) | (0.00) |
| Observations | 2754 | 2754 | 2754 | 2754 | 2754 | 2695 | 2695 | | 2695 | 2695 | 2695 |
| R-squared | 0.002 | 0.002 | 0.006 | 0.006 | 0.116 | 0.002 | 0.002 | | 0.006 | 0.007 | 0.117 |
| Adj R^2^ | 0.001 | 0.001 | 0.004 | 0.004 | 0.114 | 0.002 | 0.001 | | 0.004 | 0.005 | 0.115 |
| *p-values are in parentheses *** p<.001, ** p<.01, *p<0.05* | | | | | | | |  |  |  |  |
|  | | |  |  |  |  |  |  |  |  |  |

Regression coefficients. Outcome variable: children's c-reactive protein (log-transformed). Source: KiGGS data baseline study and wave 2. Author's own calculations.
